# Dynamic contrast-enhanced MRA of the aorta using a Golden-angle RAdial Sparse Parallel (GRASP) sequence: comparison with conventional time-resolved cartesian MRA (TWIST)

**DOI:** 10.1007/s10554-024-03259-9

**Published:** 2024-10-12

**Authors:** Camilla Giulia Calastra, Elena Kleban, Fabrice Noël Helfenstein, Fabian Haupt, Alan Arthur Peters, Adrian Huber, Hendrik von Tengg-Kobligk, Bernd Jung

**Affiliations:** 1 Department of Diagnostic, Interventional and Pediatric Radiology (DIPR), Inselspital, Bern University Hospital, University of Bern, Bern, Switzerland; 2 Translational Imaging Center (TIC), Swiss Institute for Translational and Entrepreneurial Medicine, Bern, Switzerland; 3 Swiss Cardiovascular Center, Inselspital, Bern University Hospital, Bern, Switzerland; 4 Department of Clinical Research, University of Bern, Bern, Switzerland; 5 Experimental Radiology, Department of BioMedical Research, University of Bern, Bern, Switzerland

**Keywords:** Contrast enhanced, Time resolved, MRA, Thoracic imaging, Aortic diseases, Radial trajectory, GRASP

## Abstract

**Purpose:**

To compare the application of two contrast-enhanced time-resolved magnetic resonance angiography sequences on an aortic disease patient cohort: the conventional Cartesian-sampling-based, Time-resolved angiography With Interleaved Stochastic Trajectories (TWIST) sequence, and the radial-sampling-based Golden-angle RAdial Sparse Parallel (GRASP) sequence. TWIST is highly sensitive to patient movement, which can lead to blurring and reduced sharpness of vascular structures, particularly in dynamic regions like the aorta. Such motion artifacts can compromise diagnostic accuracy. Radial-sampling-based techniques are less sensitive to motion than cartesian sampling and are expected to improve the image quality in body parts subjected to motion.

**Methods:**

30 patients (60.9 ± 16.1y.o.) with various aortic diseases underwent a 1.5T magnetic resonance angiography examination. Assessment of image quality in the ascending aorta (AA), descending aorta (DA), and abdominal aorta (AbA) on a 4-point Likert scale (1 = excellent, 4 = non-diagnostic) as well as max. aortic diameters (Dmax) were performed. T-test and multilevel mixed-effect proportional-odds models were used for the image analysis.

**Results:**

GRASP offered superior depiction of vascular structures in terms of vascular contrast for qualitative analysis (TWIST, reader 1: 1.6 ± 0.5; reader 2: 1.9 ± 0.4; reader 3: 1.1 ± 0.4; GRASP, reader 1: 1.5 ± 0.5; reader 2: 1.4 ± 0.5; reader 3: 1.0 ± 0.2) and vessel sharpness for qualitative (TWIST, reader 1: 1.9 ± 0.6; reader 2: 1.6 ± 0.6; reader 3: 2.0 ± 0.3; GRASP, reader 1: 1.4 ± 0.6; reader 2: 1.2 ± 0.4; reader 3: 1.3 ± 0.6) and quantitative analysis (TWIST, AA = 0.12 ± 0.04, DA = 0.12 ± 0.03, AbA = 0.11 ± 0.03; GRASP, AA = 0.20 ± 0.05, DA = 0.22 ± 0.06, AbA_=_0.20 ± 0.05). Streaking artefacts of GRASP were more visible compared to TWIST (TWIST, reader 1: 2.2 ± 0.6; reader 2: 1.9 ± 0.3; reader 3: 2.0 ± 0.5; GRASP, reader 1: 2.6 ± 0.6; reader 2: 2.3 ± 0.5; reader 3: 2.8 ± 0.6). Aortic Dmax comparison among the sequence showed no clinical relevance.

**Conclusion:**

GRASP outperformed TWIST in SNR, vessel sharpness, and reduction in image blurring; streaking artefacts were stronger with GRASP, but did not affect diagnostic image quality.

## Introduction

Gadolinium-based (Gd) contrast-enhanced time-resolved magnetic resonance angiography (CE-trMRA) techniques can depict the anatomy and hemodynamics of complex vascular structures [1–8]. Beyond its diagnostic capabilities, CE-trMRA emerges as a tool for monitoring disease progression over time [2, 3]. This is invaluable for making informed decisions about the timing and necessity of interventions. Furthermore, CE-trMRA plays a pivotal role in treatment planning [1, 5]. This facilitates more targeted and effective treatment strategies, enhancing overall patient care. In particular, the real-time assessment of aortic calibre, aortic aneurysms, and various aortic side branches proves paramount for diagnostic accuracy and to guide interventions focusing on individual patient needs [4]. Routine static magnetic resonance angiography (MRA) offers excellent anatomical detail but lacks the temporal resolution to always capture the optimal time point of contrast agent (CA) arrival. In abrupt changes of aortic diameters, such as in large aneurysms or complex dissections, CA distribution may be slowed significantly. Within aortic disease populations, its application holds substantial promise for advancing clinical care by delivering real-time insights into blood flow patterns. Physicians appreciate the Digital subtraction angiography (DSA) -like pattern of CE-trMRA visualisation in a similar imaging appearance [9]. DSA simultaneously offers high spatial and temporal resolution but involves invasive procedures and adds ionising radiation to patients and interventionalists. For elective cardiovascular patients, CE-trMRA inherits, therefore, a valuable alternative to DSA and computed tomography angiography (CTA), avoiding ionising radiation and providing dynamic contrast information [1–3, 5, 8]. CTA remains the reference method for aortic imaging in an acute clinical setting, typically pre-operative planning [1, 10–13] and generally pre-operative planning [14]. On the other hand, care must also be taken with gadolinium based CA (GBCA) in patients with impaired renal function.

The TWIST (Time-resolved angiography With Interleaved Stochastic Trajectories) magnetic resonance imaging (MRI) sequence is a commonly used acquisition technique to perform CE-trMRA measurements. It is based on a Cartesian acquisition with sharing k-space data between adjacent time frames to allow for a good compromise between spatial and temporal resolution [15]. However, its susceptibility to motion artefacts, such as blurring of vascular boundaries due to respiratory motion, can limit its diagnostic accuracy in dynamic imaging scenarios [16].

Acquisition techniques based on radial sampling are less sensitive to motion than those based on Cartesian sampling and consequently improve the overall image quality in the presence of motion [17]. Moreover, undersampling artefacts with radial imaging manifest as streaks, whereas they appear aliasing with Cartesian. Parallel imaging reconstruction removes aliasing from undersampled data; however, undersampling factors are limited to about 2 to 3 per phase encoding direction. In contrast to Cartesian imaging, radial undersampling artefacts appear as incoherent aliasing in multiple dimensions [18], which is well suited for 4D image reconstruction based on compressed sensing (CS) exploiting spatial and temporal correlations and using a nonlinear reconstruction to enforce sparsity in a suitable transform domain [19, 20]. A uniform k-space coverage with high temporal incoherence can be obtained for any number of views if the golden-angle rotation is applied between successive echoes [21]. This enables dynamic imaging studies using continuous data acquisition and retrospective reconstruction of image series with arbitrary temporal resolution by grouping different numbers of consecutive echoes into temporal frames. The GRASP (Golden-angle RAdial Sparse Parallel) MRI technique combines the abovementioned aspects using a stack-of-stars acquisition with a golden-angle trajectory combined with compressed sensing and parallel imaging reconstruction with high undersampling factors [22].

Previous works have successfully proven that GRASP allows for a reliable and robust assessment of dynamic CE liver imaging [23, 24] at high acceleration factors. Also, GRASP is known to provide robust imaging in situations with potential image degradation because of respiratory, cardiac, or vascular motion artefacts [25]. Because of the advantages mentioned above, GRASP has the potential to improve image quality in body regions subjected to motion and for imaging of high-flow vascular pathologies like peripheral arteriovenous malformations (AVMs) that demand high temporal resolution while maintaining a decent spatial resolution [26].

While TWIST has been adequate for many clinical applications, its limitations in handling motion artefacts may compromise diagnostic accuracy in specific scenarios, such as dynamic imaging of aortic diseases. Therefore, the objective of our intra-individual study is to rigorously compare TWIST and GRASP in terms of image quality for patients with aortic diseases. By conducting qualitative assessments of inter- and intra-observer variabilities and quantitative comparisons between TWIST and GRASP images, we aim to elucidate whether GRASP offers superior performance in these clinical contexts. This comparison is essential to identify advancements in CE-trMRA techniques that could support clinical practice by improving diagnostic precision and treatment planning in aortic pathologies.

## Materials and methods

In this cross-over single-centre prospective study, a cohort of 30 patients (60.9 ± 16.1 yo, 7 females) with various chronic aortic diseases (aortic dissection type A: 10, aortic dissection type B: 7, aortic aneurysm: 8; status after contained aortic rupture: 3, Marfan syndrome: 1, stenosis of the left subclavian artery and impact of coronary artery bypass: 1) underwent a clinical follow up routine MRI examination between July and October 2022. The standard TWIST was complemented by a GRASP sequence after the local institutional review board approved the study and all patients gave their written informed consent before the MRI examination.

### MRI data acquisition

All data were acquired on a 1.5T clinical scanner (Magnetom SolaFit, Siemens, Erlangen, Germany) equipped with a 32-channel body coil.

With each CE-trMRA acquisition, the same amount of GBCA (Gadovist 1.0 M, Bayer, Switzerland AG, Zurich) was administered (0.075 ml/(kg bw), flow rate 4 ml/s), i.e. twice during each protocol. All images were acquired during free breathing in the oblique coronal plane. To reduce bias due to contrast enhancement in the vascular systems during the second CA administration, TWIST and GRASP sequences were acquired in reverse order for half of the patients (*n* = 15), respectively. For all examinations, there was a three-minute pause between CA administrations. The reconstruction of GRASP data was performed inline at the scanner within about 30 s.

All acquisition parameters are summarised in Table 1. For the TWIST sequence, 25% of the k-space centre was used to reconstruct a time frame, whereas 33% of the remaining k-space periphery was sampled between each acquisition of the k-space centre [27]. For the GRASP sequence, 13 radial projections were used to reconstruct one-time frame.

**Table 1:** Time-resolved contrasted-enhanced magnetic resonance angiography acquisition parameters

	GRASP	TWIST
Temporal resolution [s]	1.8	1.98
Spatial resolution [mm^2^]	1.56 × 1.56	2.89 × 1.56
Slice thickness [mm]	2.5–3.5	2.5–3.5
Field Of View (FOV) [mm^2^]	400 × 400−500 × 500	400 × 400−500 × 500
Number of slices	52–56	52–56
Undersampling factor	19.1	-
Parallel Acquisition Technique (PAT)	-	2
Flip angle [°]	17	17
Matrix size	256 × 256	256 × 256
Echo Time (TE) [ms]	1.19	0.96
Repetition Time (TR) [ms]	2.6	2.04
Scan time [s]	107	213

Slice thickness, number of slices and FoV vary depending on the patient’s characteristics and are equal for the same patient for the two sequences. Spatial resolution is the acquired one without interpolation

### Qualitative image analysis

Qualitative analysis was performed using original non-subtracted 3D dynamic images. Three experienced radiologists (with 20 (#1), 5 (#2), and 8 (#3) years of experience) assessed the overall image quality independently. To this end, vascular contrast, vessel sharpness, and image artefacts of TWIST and GRASP were assessed by grading the images on a 4-point Likert scale. In detail: for overall image quality, vascular contrast, and vessel sharpness: 1 = excellent; 2 = acceptable (good); 3 = poor (still diagnostic); 4 = non-diagnostic. For image artefacts: 1 = no artefacts; 2 = minor artefacts (not interfering with diagnostic content), 3 = moderate artefacts (degrading diagnostic content, image still diagnostic), 4 = severe artefacts (non-diagnostic image).

Special attention was paid to streaking artefacts for GRASP and fold-over artefacts for TWIST when assessing image artefacts. For the overall image quality index, readers focused on vascular enhancement visibility and perfusion on the vessel of the ascending aorta, supra-aortic vessels, intercostal arteries, visceral branches, notably inferior mesenteric artery, renal cortex (primary entry and additional small communication channels in aortic dissection patients).

**Fig. 1:**
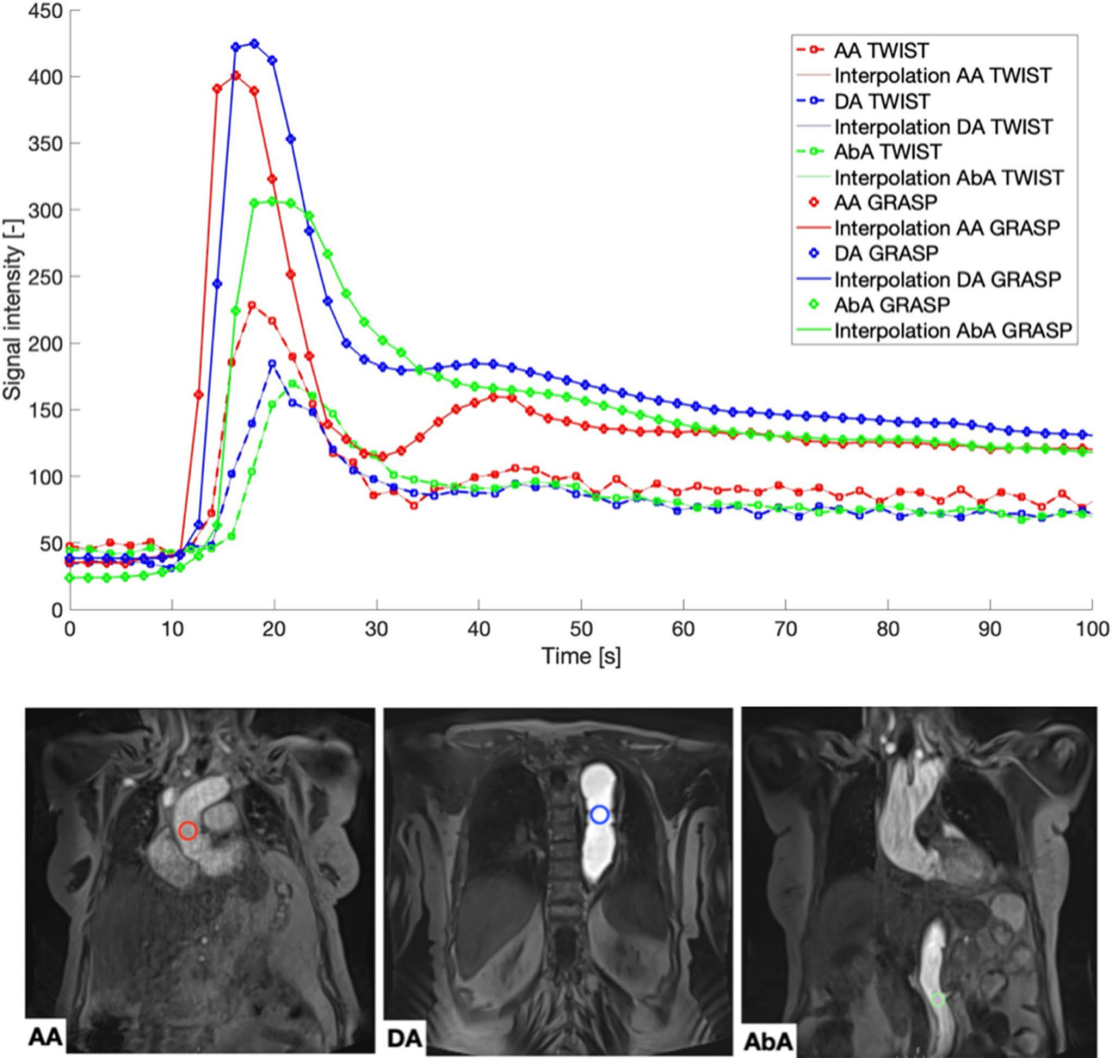
On the bottom, example of placement of ROIs at different levels of aorta: ascending aorta at the level of the pulmonary trunk, AA (red), descending aorta at the level of the pulmonary trunk, DA (blu), and abdominal aorta at the level of the renal arteries, AbA (green). On the top, plot of their signal intensity over time. Images shown are exemplary GRASP images

An experienced (20 years of practice) radiologist also assessed aortic diameters at three levels, as illustrated in Fig. 1: ascending aorta (AA), descending aorta at the level of the pulmonary trunk (DA), and abdominal aorta below the infrarenal arteries (AbA). This assessment was carried out by manually measuring the vessel diameters based on double-oblique multiplane reformation provided by clinical routine software from PACS.

### Quantitative image analysis

Quantitative image analysis was performed by MATLAB 9.12 (MathWorks, Natick, MA, USA) using original non-subtracted images. Circular regions-of-interest (ROIs) were placed at the same three aortic levels as for the qualitative image analysis (Fig. 1). In cases of an aortic dissection, the true lumen was chosen for the placement of the ROIs. When drawing circular ROIs, those slices and temporal frames were selected, where the anatomy of interest was best visible to the readers. For each patient identical ROIs at the same positions were used for both sequences. ROIs were drawn on TWIST images first for half of the patients. The temporal behaviour of the sequences was quantified by obtaining the maximum slope of the CA uptake (maxslope) and the full width at half maximum (FWHM) from the normalised and interpolated signal intensity time courses within the ROIs. Time signal intensity curves were interpolated by a factor of 10 and a smoothing function was applied to exclude the influence of noise on the outcome.

**Fig. 2:**
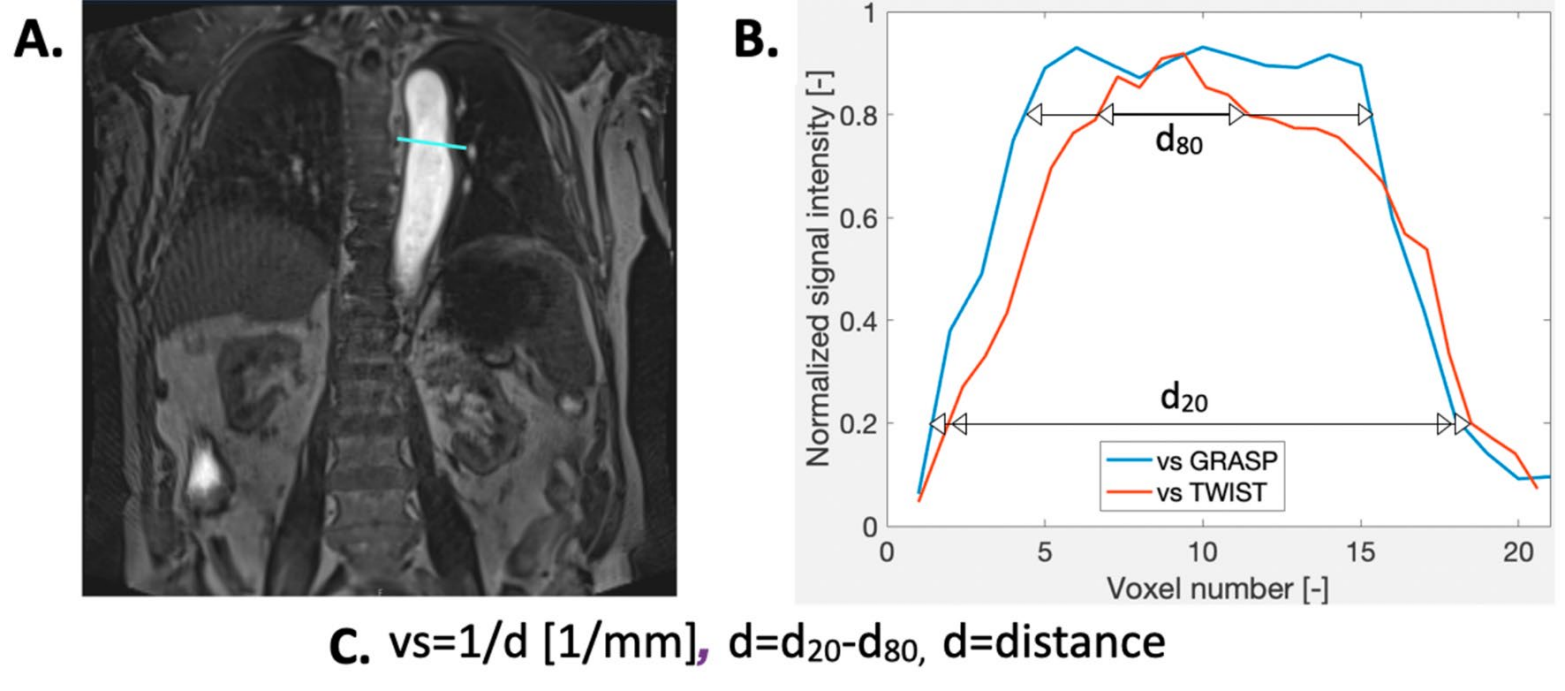
In A an example of line drawing perpendicular to the vessel, across descending aorta at the level of the pulmonary trunk, for GRASP is shown. In B there is the generation of profile assessment of vessel sharpness (vs.) from such line and a comparison with TWIST. Normalised signal intensity is represented for an easier comparison between TWIST and GRASP even if absolute signal intensity was employed for the calculus of vs. In C there is the formula used to calculate vessel sharpness from the information obtained in B

Spatial blurring was quantified by calculating vessel sharpness as follows: at the same levels of the aorta as described above a straight line perpendicular to the vessel wall was drawn across the aorta. Vessel sharpness (*vs.)* of the boundary was then calculated from the resulting signal intensity profile as vs. = 1/d. The value d (millimetres) is the distance between those points on the drawn line, between which the signal intensity changed from 20 to 80% of the absolute intensity difference, i.e. the difference between the maximal and the minimal signal intensity values [28] (Fig. 2).

Signal-to-Noise ratio (SNR) calculation was performed by adopting the first method described in the previous work [29]. Images from the second and the third time frames (before the bolus onset) were subtracted for noise. The first time frame was not used as it has slightly different acquisition parameters for TWIST.

### Statistical analysis

Statistical analysis was performed using R, version R 4.2.2 (R Foundation for Statistical Computing, 2021) and Excel, version 16.66.1 (22101101), (Microsoft, Redmond, Washington, USA). The statistically significant difference for all tests is set to *p* < 0.05.

T-tests were used for the quantitative image analysis and for the evaluation of diameter measurements. Multilevel mixed-effect proportional-odds models were used for the qualitative analysis. Multilevel mixed-effect proportional-odds models include the scores as an ordinal dependent variable, the sequence (GRASP vs. TWIST) as the fixed factor, and patient pseudo-ID and reader ID as random factors. The link function is logit (proportional odds). With such models, positive estimates indicate higher metrics for GRASP than for TWIST, suggesting better image quality or performance of TWIST over GRASP. On the contrary, negative values indicate higher metrics for TWIST than for GRASP, suggesting better image quality or performance of GRASP over TWIST.

## Results

All measurements could be successfully performed in all patients. The results reveal that GRASP demonstrates higher SNR and overall image quality, whereas more artefacts based on radial undersampling were observed compared to Cartesian TWIST.

**Fig. 3:**
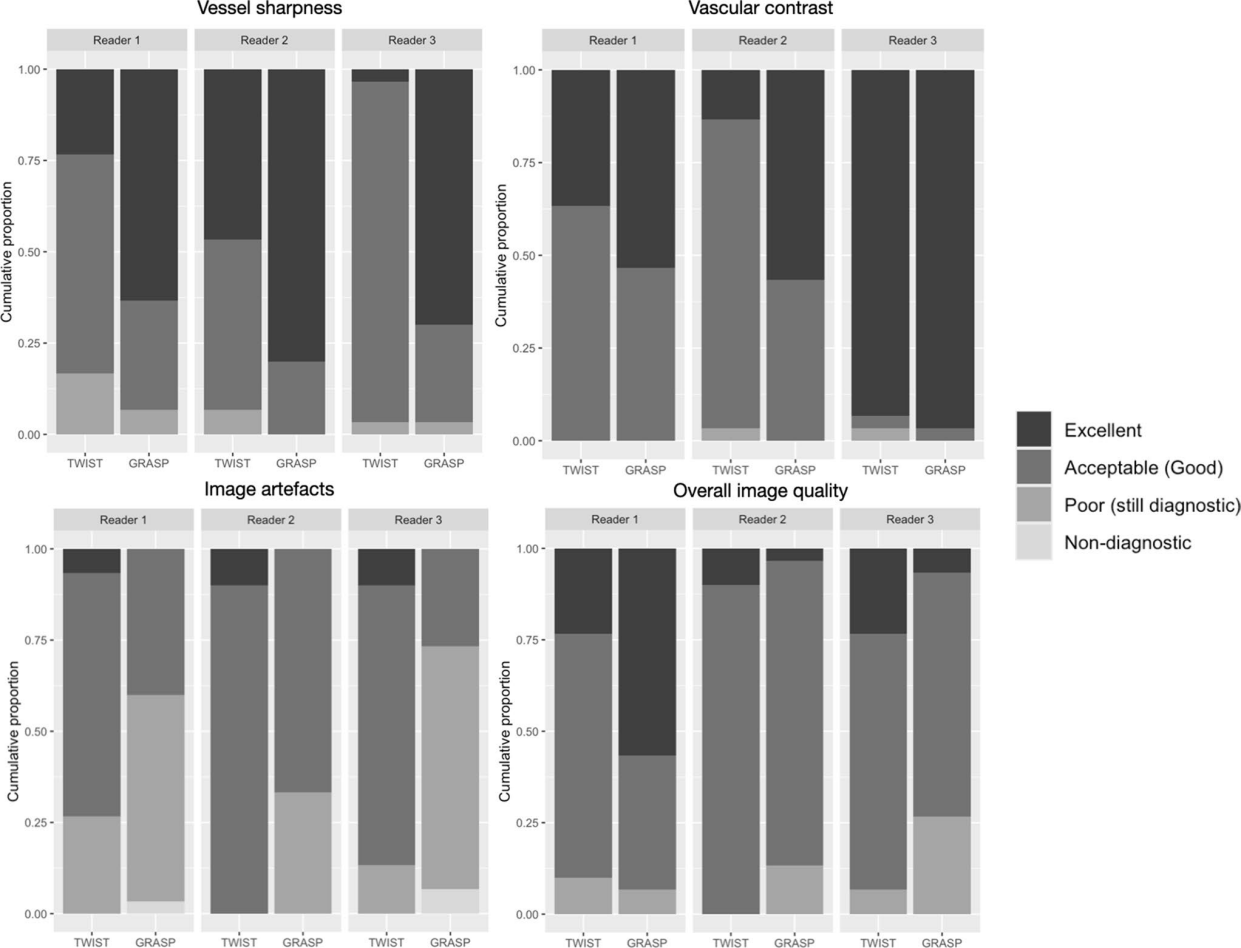
Stacked bars of qualitative assessment scores among the two sequences under analysis. 4-point Likert scale used: 1 = excellent; 2 = good; 3 = poor; 4 = non-diagnostic

Figure 3 reports lower scores with GRASP than with TWIST, meaning better performance of GRASP over TWIST in terms of vascular contrast and vessel sharpness. Our statistical analysis confirms these results (negative model estimates; all *p* < 0.05; Table 2). However, TWIST is superior to GRASP (scores are higher with GRASP than with TWIST; Fig. 3) for image artefacts (positive model estimates; *p* < 0.05; Table 2). Even if severe image artefacts are characteristic of the data acquired using the GRASP sequence, these do not influence the diagnostics as corroborated by the image quality index (*p* = 0.05). The inter-reader analysis indicates that readers do not agree more than would be expected if the rating was merely random (Table 3).

**Table 2:** Results of the multilevel mixed-effect proportional-odds cumulative logit models

	Estimates	*p*-value
Vessel sharpness	−2.3	*p* < 0.05
Vascular contrast	−1.5	*p* < 0.05
Image artefacts	2.7	*p* < 0.05
Image quality	0.3	*p* = 0.05

Quantitative comparison of image quality metrics between TWIST and GRASP sequences. Positive estimates indicate higher metrics for GRASP than for TWIST, suggesting better image quality or performance of TWIST over GRASP. On the contrary, negative values indicate higher metrics for TWIST than for GRASP, suggesting better image quality or performance of GRASP over TWIST

**Table 3:** Inter-rater qualitative results. Comparison of the image quality scores among readers through Fleiss’ Kappa

	Fleiss’kappa		*p*-value	
	TWIST	GRASP	TWIST	GRASP
Vessel sharpness	−0.18	0.03	0.08	0.70
Vascular contrast	−0.15	0.01	0.13	0.91
Image artefacts	0.16	0.10	0.05	0.32
Image quality	0.00	−0.01	0.97	0.24

The signal intensity values (Fig. 1) obtained with the GRASP sequence at all aorta locations are higher than those obtained with the TWIST sequence. In addition, the corresponding time courses are smoother as visible by fewer signal fluctuations compared to TWIST, in particular in later time frames.

**Fig. 4:**
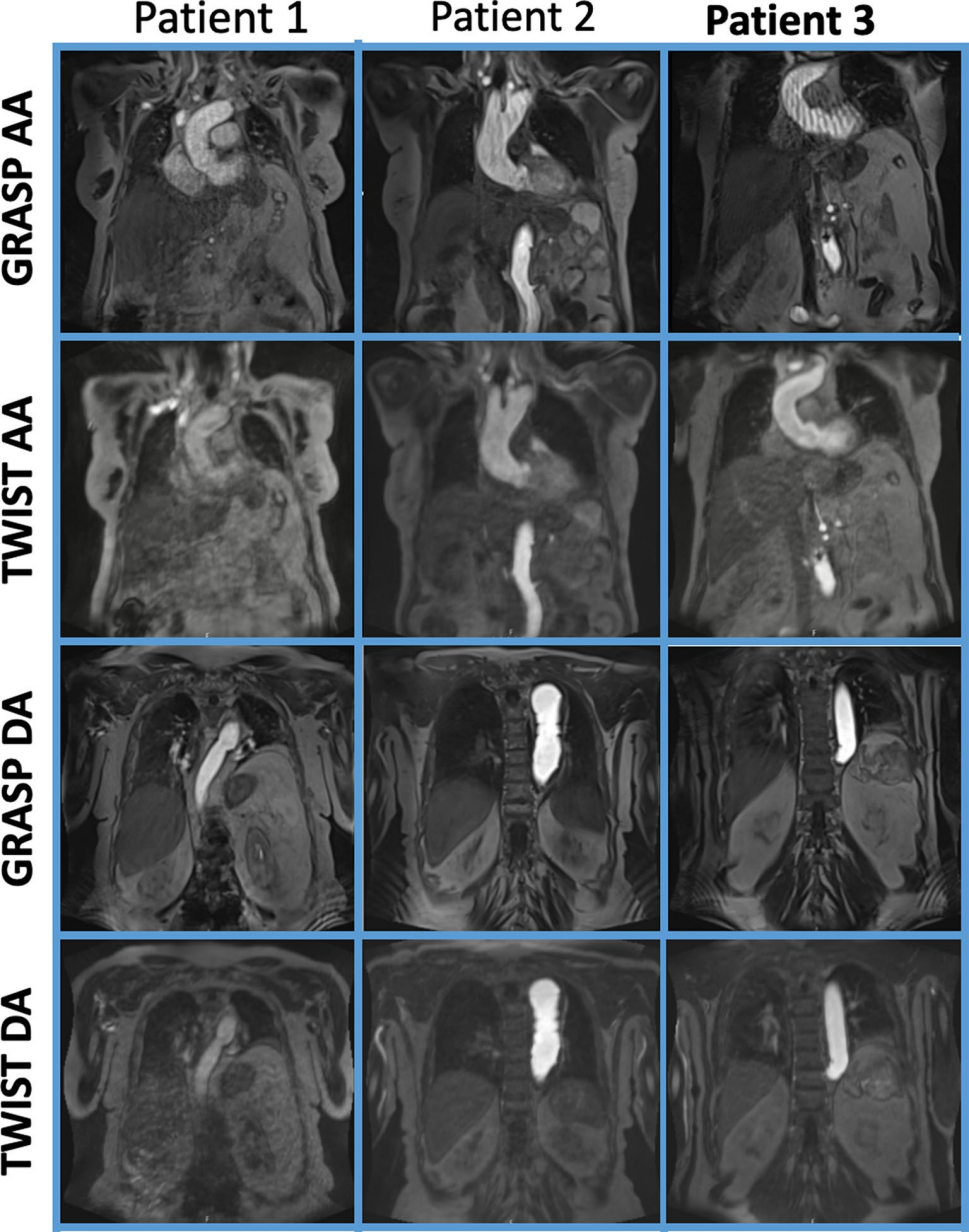
Representative image comparison between GRASP and TWIST FoV = 400 × 400 for three patients at ascending aorta (AA) and descending aorta (DA) at the level of the pulmonary trunk. On the top line images of GRASP from three patients of the study show AA being affected by streaking artefacts, in an increasing amount from left to right. On the third line images of GRASP show DA not being affected by artefacts. We did not report images of abdominal aorta because for more than half datasets the images would be the same as the ascending aorta. GRASP images are characterised by more pronounced artefacts and by an improved vascular contrast and vessel sharpness compared to TWIST

Three patient examples with streaking artifacts corresponding to the artifact level 2, 3, 4 as defined in the Likert scale for the qualitative analysis are shown in Fig. 4. Only the images of the AA and the DA are shown since the AbA is close to the location of the AA image. Overall, the highest amount of artefacts were observed in the AA.

**Fig. 5:**
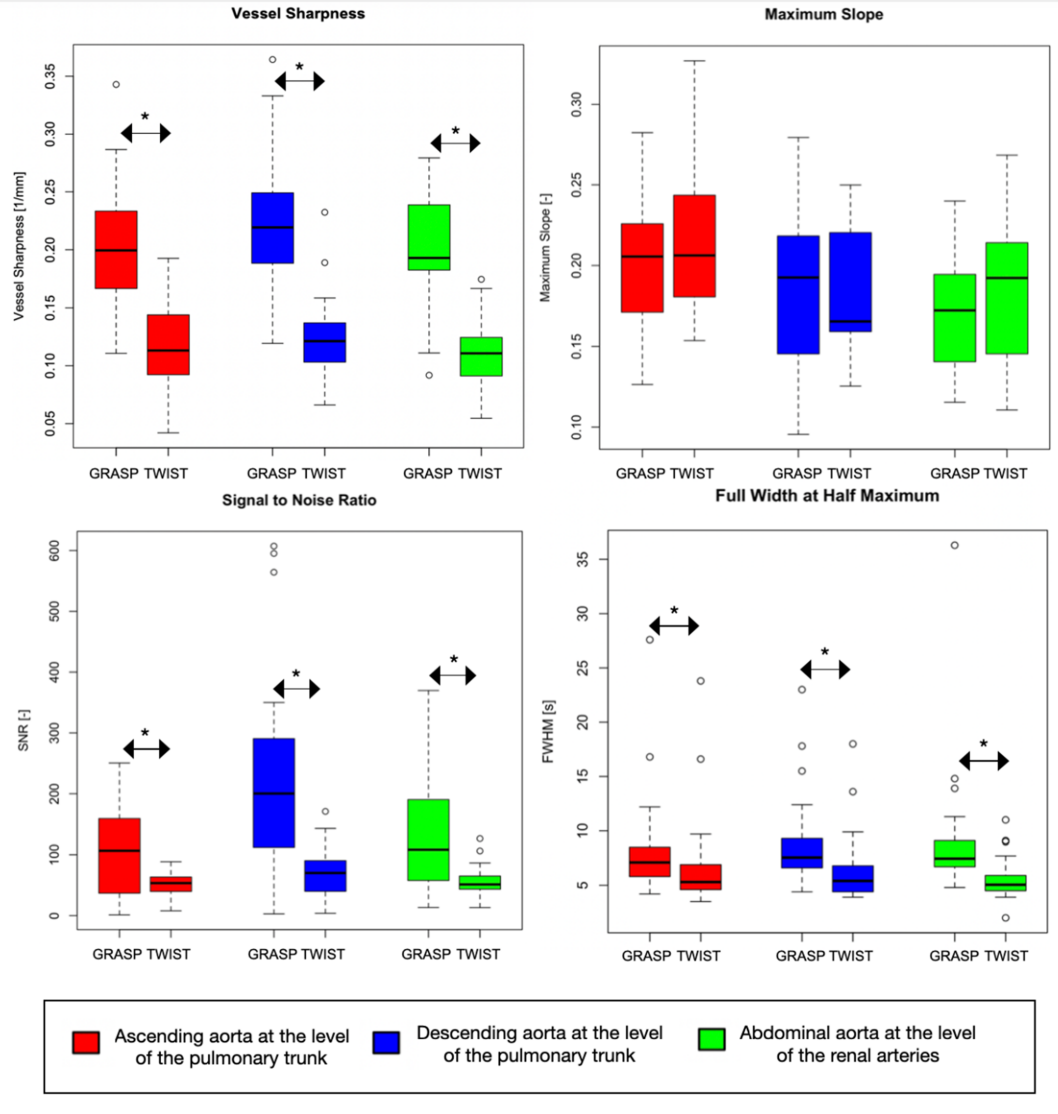
Quantitative results at ascending aorta (AA), descending aorta (DA) at the level of the pulmonary trunk and abdominal aorta at the level of the renal arteries (AbA) for GRASP and TWIST. The star means a significant statistical difference between the two sequences

In contrast, the TWIST sequence is characterised by a blurring of sharp contrast or object edges (Fig. 4). In the same Figure, the tissue contrast appears somewhat different in the GRASP sequence compared to TWIST. As shown in Fig. 5, this difference is also marked by the quantitative results, as GRASP sequence achieved superior values in vessel sharpness and SNR. FWHM was better on images produced with the TWIST sequence, and there are three outliers for both sequences. The maximum slope of the CA uptake was similar for both sequences.

In addition to these findings, a comparison of the measured diameters (D1, D2, D3) between the GRASP and TWIST sequences revealed statistically significant differences. The mean diameters were consistently larger with the TWIST sequence compared to GRASP, as reported in Table 4.

**Table 4:** Diameter evaluation results. Table showing the manually measured diameters of the ascending aorta (D1), descending aorta at the level of the pulmonary trunk (D2), and abdominal aorta at the level of the infrarenal arteries (D3)

	D1 [mm]	D2 [mm]	D3 [mm]
TWIST	33.5 ± 3.9	31.1 ± 7.4	23.90 ± 5.8
GRASP	33.0 ± 3.9	30.5 ± 7.4	22.9 ± 5.9
*p*-value	*p* < 0.05	*p* < 0.05	*p* < 0.05

## Discussion

This intra-individual prospective study focused on evaluating a golden-angle (GA) radial CE-trMRA sequence as an alternative to the conventional Cartesian sequence for imaging the aorta in patients with aortic diseases. We chose the standard TWIST sequence for aortic imaging due to a lack of specific guidelines. We adjusted the GRASP sequence with comparable parameters, believing that minor variations would not significantly impact the study’s outcome. Our results suggest that the GRASP sequence improved the image quality of the aorta despite the presence of streaking artefacts (with its current temporal resolution), which agrees with a previous study [20]. As shown by the quantification of vessel sharpness and SNR, the depiction of the aorta is superior to conventional TWIST imaging. This is corroborated by the scoring results of the experienced radiologists. A very moderate low-pass filtering in the temporal domain across the areas of interest has been observed for GRASP, whereas the uptake of GBCA described by the maximum slope is comparable in the two sequences.

### Imaging artefacts

The artefact level is significantly higher in the GRASP images. This is caused by streaking artefacts as a result of the high level of undersampling [20]. Using more radial projections may reduce the undersampling factor and mitigate the streaking artefacts, although at a cost of lower temporal resolution. Therefore, adapting the number of radial projections for the required temporal resolution for each application is beneficial for image quality; e.g. a somewhat lower temporal resolution may be sufficient for aortic applications while resulting in a lower level of streaking artefacts. Streaking artefacts are more pronounced in the periphery of the FoV than the centre of the FoV. The ascending aorta and the aortic arch are most affected within the region of interest. The difference in streaking artefacts among patients and aorta levels is assumed to be due to complementary factors such as coil configuration [20], the pronounced signal change in the early arterial phase, and the presence of implants (TEVAR, sternal clips or cerclage).

Furthermore, while high temporal resolution (~ 2s) can be essential for capturing rapid hemodynamic changes, it might not be optimal for aortic imaging. Thus, a somewhat lower temporal resolution may still provide adequate diagnostic information while reducing the level of streaking artefacts, thereby improving the overall image quality. Thus, balancing temporal resolution and image quality is crucial and may differ depending on the type of application.

As for artefacts observed in the TWIST sequence, blurring of sharp contrast or object edges is due to the lower resolution in phase encoding direction and its Cartesian nature [30].

### Spatial appearance

Improved vessel sharpness, qualitatively and quantitatively, for all ROI locations in GRASP compared to TWIST is assumed to result from the higher in-plane resolution and the reduced sensitivity of the radial trajectory to breathing motion. In addition, the image sharpness of the GRASP sequence is influenced by the choice of density compensation function for the balance of the k-space energy content and by the spatial weight in the CS reconstruction [31, 32]. However, this cannot be controlled due to a standardised vendor reconstruction.

The comparison of the diameters measured with the GRASP and TWIST sequences revealed that GRASP consistently produced significantly lower values across all measured locations (D1, D2, D3). However, the differences between the mean values are in the order of maximum 1% for each location, which is supposed to be in the range of the variances drawing the ROI. Furthermore, even though significant such small differences do not have a clinical impact on therapy decision. While we do not have a definitive “ground truth” to confirm which measurements are more accurate, it is plausible to trust the data obtained with the GRASP sequence, given its higher spatial resolution. The slightly larger diameters with TWIST could be the impact of partial volume effects caused by the larger voxel size. This effect can lead to an overestimation of vessel diameters, particularly in regions where the vessel edges are less sharply defined.

### Temporal behaviour of the contrast enhancement

The analysis of the temporal behaviour of both sequences revealed a significantly lower FWHM of the first-pass bolus for TWIST in all locations compared to GRASP, despite the higher temporal resolution for GRASP and the shared view of TWIST that can generate temporal blurring [33]. This may be a result of the shorter acquisition of the k-space centre (scan time for the 25% of k-space centre used to reconstruct one time frame ~ 1s) compared to GRASP, where k-space centre crossing spokes (during the temporal resolution of 1.8s) were used to reconstruct a time frame. Furthermore, the CS reconstruction of the GRASP sequence includes a temporal regularisation term, which may introduce temporal blurring. However, it turned out by the qualitative analysis of vascular contrast that the observed differences in the dynamics are not of clinical relevance. Interestingly, two outliers for the FWHM values at all aorta locations are observed. A more detailed assessment of these data revealed abnormal flow direction in these patients due to the compressed true lumen of the aorta. No significant differences between sequences were found for the maximum upslope of the first-pass bolus. Additionally, the results of FWHM and max slope may not have direct clinical relevance but corroborate some clinical observations and might be relevant for quantification of some aspects [34] and hence could be useful for clinicians. For example, the quantification of aspects of vascular malformations would be of interest. We did not perform quantifications, e.g. in aortic root or ascending aorta, because these MR acquisitions are non-triggered and there is too much motion.

### Signal-to-noise ratio

SNR is highly affected by acquisition and reconstruction parameters. TWIST data were acquired with parallel imaging, resulting in a spatially dependent SNR penalty due to g-factor-based noise enhancement [35]. The noise behaviour of GRASP strongly depends on the choice of the regularisation parameters in the spatial and temporal domain for the CS reconstruction [36]. Nevertheless, with a parallel imaging factor commonly used for TWIST protocols and the standardised CS reconstruction parameters, a higher SNR was reached for the GRASP sequence despite its higher spatial resolution compared to TWIST imaging.

### Limitations

The quantitative analysis was confined to three specific regions within the aorta, which might not fully represent other areas or side branches, such as aortic side branches. In addition, due to the heterogeneity of the patient cohort, aortic calibre, the extent of the dissection flap in aortic dissection, or the integrity of various aortic side branches have not been assessed. We focused on overall image quality and larger vessels, as spatial resolution limits and motion artefacts prevented a dedicated assessment of small vessels like intercostal and renal arteries.

Furthermore, there is no ground truth to compare with, rendering the visual assessment of certain parameters more difficult. We observed a general lack of agreement among readers observed. Such variance could be attributed to insufficient training sessions for the radiologists involved. To mitigate this issue in future studies, it is advisable to conduct more extensive training sessions. These sessions could involve reviewing multiple images corresponding to each Likert scale point, thereby facilitating the achievement of consensus among radiologists. Moreover, enhancing the study’s objectivity could be achieved by identifying less subjective endpoints or directly inquiring more details about the diagnostic nature of the image. By incorporating less subjective criteria or explicitly assessing diagnostic relevance, the study’s subjectivity could be reduced, leading to more consistent results.

### Outlook

The optimisation of regularisation parameters in the CS reconstruction [31] in the spatiotemporal domain tailored for this specific GRASP application may allow for finding a better compromise between SNR, vessel sharpness, temporal blurring, and streaking artefacts. Using an improved CS-type reconstruction [32] may yield reduced undersampling artefacts. However, a systematic investigation of such recon parameters was beyond the scope of this work.

## Conclusions

In conclusion, we quantitatively and qualitatively compared the performance of a Cartesian and radial sequence for CE-trMRA on a cohort of patients with aortic diseases. GRASP outperformed TWIST offering superior SNR and image quality, comprising vessel sharpness, vascular contrast, and image artefacts. Streaking artefacts were more pronounced on GRASP images but did not affect diagnostics. The findings suggest that GRASP has the potential to provide reliable imaging of the aorta (or where organ motion is likely to degrade image quality) in serial follow-up, which is essential in clinical decision-making. The observations also indicate the need for more detailed investigations in the future to optimise the CS reconstruction parameters in the spatial and temporal domain, namely the undersampling factor affecting the temporal resolution as well as the regularisation in the spatial and temporal domain, for such CE-trMRA application.

## Data Availability

Data are available at request.

## Abbreviations

AA: Ascending aorta
AbA: Abdominal aorta
AVM: Arteriovenous malformation
CA: Contrast media
CE-trMRA: Contrast-enhanced time-resolved magnetic resonance angiography
CS: Compressed sensing
CTA: Computed tomography angiography
DA: Descending aorta
DSA: Digital Subtraction Angiography
FoV: Field of view
FWHM: Full width at half maximum
GA: Golden angle
Gd: Gadolinium
GBCA: Gadolinium based contrast agent
GRASP: Golden-angle RAdial Sparse Parallel
maxslope: Maximum slope of the contrast agent uptake
MRI: Magnetic resonance Imaging
PAT: Parallel acquisition technique
ROI: Region of interest
SNR: Signal-to-Noise ratio
TWIST: Time-resolved angiography With Interleaved Stochastic Trajectories
vs: Vessel sharpness
